# Genome-wide recombination map construction from single individuals using linked-read sequencing

**DOI:** 10.1038/s41467-019-12210-9

**Published:** 2019-09-20

**Authors:** Andreea Dréau, Vrinda Venu, Elena Avdievich, Ludmila Gaspar, Felicity C. Jones

**Affiliations:** Friedrich Miescher Laboratory of the Max Planck Society, Max-Planck-Ring 9, 72076 Tübingen, Germany

**Keywords:** Meiosis, Computational biology and bioinformatics, Evolution, Genetics, Molecular biology

## Abstract

Meiotic recombination rates vary across the genome, often involving localized crossover hotspots and coldspots. Studying the molecular basis and mechanisms underlying this variation has been challenging due to the high cost and effort required to construct individualized genome-wide maps of recombination crossovers. Here we introduce a new method, called ReMIX, to detect crossovers from gamete DNA of a single individual using Illumina sequencing of 10X Genomics linked-read libraries. ReMIX reconstructs haplotypes and identifies the valuable rare molecules spanning crossover breakpoints, allowing quantification of the genomic location and intensity of meiotic recombination. Using a single mouse and stickleback fish, we demonstrate how ReMIX faithfully recovers recombination hotspots and landscapes that have previously been built using hundreds of offspring. ReMIX provides a high-resolution, high-throughput, and low-cost approach to quantify recombination variation across the genome, providing an exciting opportunity to study recombination among multiple individuals in diverse organisms.

## Abstract (web summary)

Variation of recombination rates within genomes has important implications in genetics and evolution. Here, the authors develop a method for building genome-wide recombination maps from single individuals using linked-read sequencing data, and report its application in mouse and stickleback fish.

## Introduction

Recombination is an essential process during meiosis. Chromosome segregation often occurs through crossing-over, which involves reciprocal exchange among homologous chromosomes and plays an essential role in meiotic chromosome segregation in sexually reproducing organisms. By shuffling parental alleles to produce novel haplotypes it is also a key source of genetic diversity that has considerable implications for the genomic landscape of variation and the evolutionary process.

In most diploid organisms, recombination is functionally constrained by the necessity for at least one recombination event per homologous chromosome pair (this ensures proper segregation during Meiosis I)^1^. Defective, excessive, or deficient recombination can cause inviable gametes and developmental abnormalities^2,3^. For these reasons the number of crossovers and their genomic locations are thought to be tightly regulated and highly constrained^4^.

Despite this core functional constraint, recent studies have revealed remarkable variation in recombination at multiple different scales (between and along chromosomes, among individuals, sexes, populations, and species/taxa)^5–12^. Crossovers are not uniformly distributed across the genome and the frequency (recombination rate), can vary by orders of magnitude and involve genomic hotspots and coldspots. For example, a well-studied recombination hotspot (*Hlx1*) on mouse chromosome 1 has a remarkably high recombination rate of 2.63 cM within a narrow 2.8 kb interval in F1 hybrid male mouse (C57BL/6J x CAST/EiJ), yet is relatively colder in females of the same background and among other strains^8^. This among strain variation is partly attributable to the strain genotype at the trans-acting recombination modifier protein PRDM9. Conversely recombination coldspots with a lack of crossovers in genomic regions as large as 41 Mb have also been reported^13,14^.

Part of the extensive variation in recombination among organisms may stem from the impact of recombination on individual fitness and rates of adaptation in natural populations—in addition to its fundamental role in meiosis, recombination impacts the inheritance of linked alleles, and its modifiers may be subject to different selection pressures in different populations and taxa. Depending on the evolutionary context, recombination may be beneficial if it breaks down linkage between deleterious and beneficial alleles (known as the Hill–Robertson effect^15,16^), or deleterious if it breaks linkage between two adaptive alleles^17^.

With the knowledge that number and genomic location of recombination can influence the segregation of traits, fitness of an organism, and adaptation in natural populations, there is increasing interest in the fields of medicine, agriculture, and evolutionary genomics in the empirical quantification of fine-scale variation in recombination among individuals, populations, and species. Despite diverse approaches (linkage-maps, high density genotyping of pedigrees, and individual sperm typing/sequencing), empirically quantifying recombination variation within and among individuals remains a challenge due to the expense and data intensity required to build numerous individualized genome-wide maps of recombination rate^8,12,18–25^. Other less data intensive approaches, such as comparisons of recombination among taxa using statistical estimates of recombination from population genetic (polymorphism) data, provide population and sex-averaged historical estimates of recombination rate and can be confounded by differences in the demographic history of the taxa and differences in the effective population size of the local genomic regions being compared. Further, these averaged estimates make genetic dissection of molecular mechanisms underlying recombination variation difficult. In this study, we address these challenges by introducing a new and powerful low-cost method that quantifies empirical recombination events across the genome of a single individual using linked-read sequencing of gametes.

Linked-read libraries are generated from long (high molecular weight (HMW)) DNA molecules using a 10X Genomics Chromium controller. Numerous short reads are produced from DNA molecules encapsulated inside nanoliter-sized droplets. Using their droplet-specific barcode these short reads can be computationally reconstructed into single molecules after Illumina sequencing. This low-cost long-range information can be used to solve the problem of haplotype determination. Our pipeline called ReMIX mines the long-range information in linked-read data to identify recombination crossovers across the genome. ReMIX makes use of some parts of the 10X Genomics pipeline, Long Ranger^26^, but deviates from it in a number of important ways. Long Ranger aligns reads to a reference sequence, calls and haplotype phases SNPs, reconstructs molecules, and identifies indels and large-scale structural variants. It makes use of molecules that have a high probability of assignment to only one haplotype phase. Molecules that contain reads of mixed haplotype assignment (some reads assigned to one haplotype while others are assigned to the alternate haplotype), are considered to be errors and are discarded. However, when sequencing linked-read libraries from gamete DNA these haplotype switching molecules can also represent a valuable fraction of molecules spanning meiotic recombination crossovers. ReMIX identifies these valuable molecules and is the first method to enable reconstruction of individualized genomic recombination landscapes using linked-reads.

The linked-read information is exploited by ReMIX during three steps: identification of high-quality heterozygous variants, reconstruction of molecules, and the haplotype phasing of each molecule. The molecules identified as recombinant are then used to build an individualized genomic map of recombination crossovers, enabling us to quantify recombination variation across the genome.

We demonstrate our method using gametic tissue from a hybrid mouse (*Mus musculus domesticus* × *Mus musculus castaneus*) and a stickleback fish (*Gasterosteus aculeatus*). Genetic maps, available for both organisms, allow us to evaluate the accuracy of ReMIX. To validate the precision of our pipeline, we also use samples from the somatic tissue of the tested individuals as a negative control, as well as simulated data to determine the sensitivity and specificity of our method in genomes with different levels of polymorphisms. Using data from only a single individual and without prior knowledge of polymorphic sites, ReMIX obtained results that follow the same pattern of the previously described recombination maps, but with considerably higher resolution of the detected crossovers and lower costs compared to previous methods.

## Results

### Linked-read sequencing of pools of gametes

The novel method and algorithm that we present in this study uses pooled gamete DNA as starting material and reliably identifies recombination landscape of an individual at the whole genome level. Here we report the complete pipeline and results obtained by applying our method to an individual C57BL/6Ncrl × CAST/EiJ hybrid mouse and freshwater stickleback fish. HMW DNA (>40 kb) was extracted from purified sperm cells and somatic tissue of both mouse and fish individuals (spleen and kidney, respectively). 10X Genomics linked-read genomic libraries were prepared on a Chromium controller and the resulting linked-read libraries were sequenced on an Illumina HiSeq3000 sequencer. Reads obtained from the sequencer were then processed through our ReMIX pipeline to identify recombinant molecules and quantify the genomic recombination landscape of each individual.

### Overview of the ReMIX algorithm

**Fig. 1:**
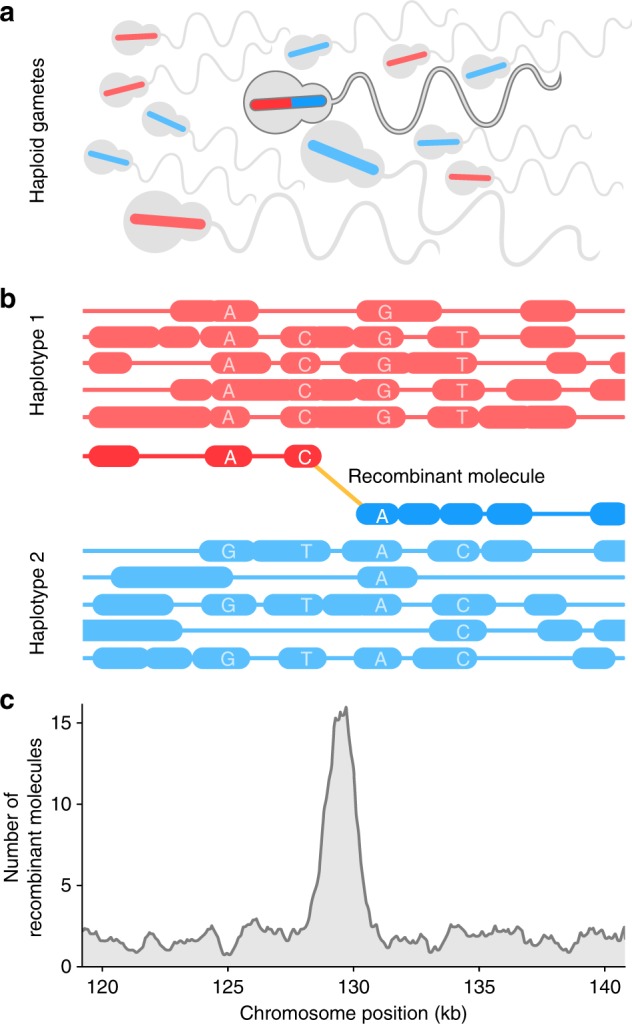
Construction of individualized genomic recombination maps using ReMIX. **a** DNA is isolated from a pool of sperm where each cell represents a haploid product of a single meiotic event. Sperm with recombinant chromosomes are shown carrying bars colored both red and blue, while non-recombinant chromosomes are shown as solid red or blue. **b** ReMIX identifies high-quality heterozygous variants, reconstructs molecules, then determines their haplotype phase. Three categories of molecules are identified: those belonging to haplotype 1 (red), haplotype 2 (blue), and recombinant molecules that switch from one haplotype to the other. Each contiguous line represents a molecule with the linked-reads marked by thick blocks. **c** Identified recombinant molecules are used to quantify the recombination rate across the genome

ReMIX requires linked-reads generated from haploid gamete DNA as input. From meiotic division, a haploid gamete comprises of a single copy of each chromosome in the genome—products of reductional cell division that are recombinants of the diploid parental chromosomes. Of the millions of linked-read molecules sequenced, the majority will be assigned with high probability to one of the two parental haplotypes. A small fraction of molecules (those spanning recombination crossovers) will contain reads that switch between the haplotypes (Fig. 1a). The role of ReMIX, after filtering and phasing, is to identify the rare fraction of recombinant molecules as those that switch between haplotypes (Fig. 1b). For this, our pipeline aligns the linked-reads to a reference genome sequence in order to identify high-quality heterozygous variants and to reconstruct the original molecules. After phasing the variants using the molecule information, the phase of each molecule is computed based on its reads spanning heterozygous-phased variants. Since the total number of sequenced gametes is high and the resulting per base coverage is high, the read coverage of each individual molecule can be considerably lower without compromising performance (<0.5×). Thus, the correct phasing of a maximum number of molecules by ReMIX is a function of the ratio between the density of heterozygous variants in the focal individual and the number of reads per molecule. In the end, the identified molecules are separated into those that are entirely non-recombinant (haplotype 1 or 2 molecules), or alternatively, recombinant (haplotype switching) molecules (full details in the “Methods” section).

A haplotype switching molecule may be generated from a true recombinant molecule or alternatively represent a false positive caused by bioinformatic errors, such as sequencing error, incorrect read mapping, structural variation, or barcode sharing among molecules from the same part of the genome. Our pipeline therefore incorporates several filtering steps to remove false-positive recombinant molecules. ReMIX initially filters the linked-reads based on the barcode sequence and the quality of the read. After variant calling the variants are filtered to remove polymorphisms showing allelic bias, and after molecule reconstruction, molecules with extreme high or low coverage are removed. Finally, after the haplotype phasing of molecules, genomic regions that are not covered by a similar number of molecules for each haplotype are removed. These filters allow us to remove the regions that can introduce errors in the mapping or the phasing, such as copy number variation, small deletions, inversions, translocations, etc. Finally, the ReMIX pipeline identifies molecules that have a high probability of containing a real crossover (e.g. stickleback mean probability 0.982 ± 0.068SD, source data provided as a Source Data file) along with the genomic position of that crossover.

By considering the quality of each base within a molecule, requiring at least three variants representing each haplotype, and ≥70% of reads on each side of a switch phased to the correct haplotype, ReMIX allows small erroneous switches in haplotype state within a recombinant molecule caused by single read low-quality base calls. Information on the location of recombinant molecules is then used to build an individualized genomic map of recombination crossovers (Fig. 1c).

### Identification of known hotspots in mouse

The genomic recombination landscape is well studied in various laboratory mouse strains, with one of the highest resolution sex-specific recombination maps constructed in Paigen et al.^8^. Focusing on chromosome 1, the authors genotyped 6028 progenies produced from C57BL/6J × CAST/EiJ and CAST/EiJ × C57BL/6J hybrids, mapped the locations of 5742 crossover events, and revealed the presence of a number of highly localized sex-specific recombination hotspots^8^. To evaluate the performance of our ReMIX pipeline, we analyzed linked-read libraries produced from the sperm, and as a negative control, somatic tissue from the spleen, of a single C57BL/6Ncrl × CAST/EiJ hybrid male. We then compared ReMIX results with the high-resolution recombination map from the 1479 C57BL/6J × CAST/EiJ male progeny^8^.

Whole genome linked-read libraries were generated from sperm and somatic cells in order to sample a similar number of recombinant molecules on chromosome 1 as reported in Paigen et al.^8^. We prepared six parallel reactions using the 10X Genomics Chromium controller—each with ~1.2 ng of DNA, approximately corresponding to a total of ~1700 haploid genomes. The final libraries were selected for an average of 600 bp insert size and sequenced at 170× coverage with 2 × 150 bp paired reads on an Illumina HiSeq3000 giving an expected read coverage per individual molecule of ~0.1×. Both sets of linked-reads were analyzed using ReMIX and the latest version of the mouse reference genome, NCBI Build 38 (mm10) [GCF_000001635.20].

A crude estimate of the expected number of recombinant versus non-recombinant molecules can be made: for linked-read libraries made from a single gamete with an average molecule size of 60 kb, sex-averaged map lengths of ~1630 cM (genome-wide) and 96.55 cM (chromosome 1)^27^, and assembled genome size of 2.9 Gb, we might expect to find recombinant molecules spanning crossovers at a frequency of 3.3 × 10^−4^ and 1.8 × 10^−5^, respectively (16.3 and 0.9 recombinant molecules in a genome-wide total of 48,333 molecules from a single gamete). In a pool of 1700 gametes (equivalent to the number of gametes sequenced here), we expect to uncover 27,710 recombinant molecules across the genome, with roughly 1641 of these located on chromosome 1.

**Fig. 2:**
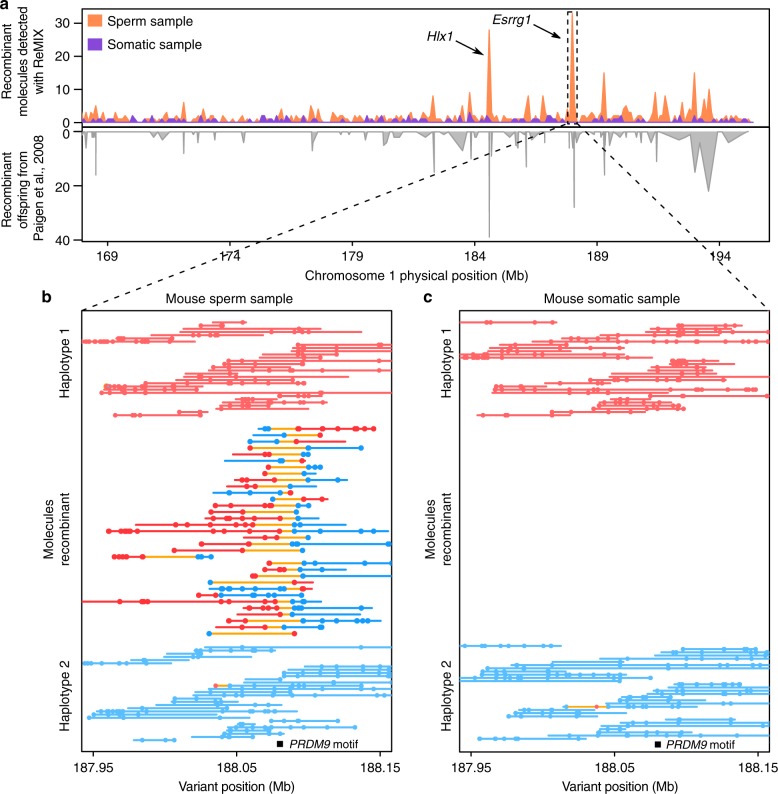
ReMIX correctly detects fine-scale recombination variation and hotspots on mouse chromosome 1. **a** The recombination rate on the south end of chromosome 1 (169–195.4 Mb, mm10), determined by ReMIX (orange above axis) corresponds well to the rate described in Paigen et al.^8^ (gray below axis). As a negative control, somatic tissue (purple) shows a minimal number of dispersed recombinant molecules. **b** The three types of molecules identified by ReMIX in the sperm sample in the region of a well-known recombination hotspot (*Esrrg1*,^8,28^). Each line represents a single molecule and each dot a high-quality heterozygous variant phased as haplotype 1 (red) or haplotype 2 (blue). Joining lines represent the inferred phase of the molecule with orange lines indicating a switch between haplotype states. For graphical reasons, we represented all the recombinant molecules detected by ReMIX but only 30 random (classical) molecules for each haplotype. **c** The corresponding region for somatic tissue lacks recombinant molecules. PRDM9 plays a role in initiating crossovers at the *Esrrg1* hotspot and has a DNA-binding motif (black bar) located near the midpoint of the detected recombinant molecules

After stringent filtering of the sperm sample ReMIX retained 1210M reads and reconstructed 148M molecules with an average of eight linked-reads per molecule. A total of 30,508 (0.02%) molecules were identified as recombinant (genome-wide) and 2369 of these were located on chromosome 1. Crossover positions of the recombinant molecules cluster into hotspots in a pattern closely mirroring the previously described male recombination map^8^ both in terms of position and intensity (Fig. 2a and Supplementary Fig. 2).

Accounting for false positives (see below), we see a number of windows that have significantly more crossovers than expected by chance (Wilcox rank sum test, *p* < 9.72 × 10^−20^), suggesting the presence of hotspots in the mouse genome. In contrast, recombinant molecules detected in the somatic sample are less frequent, have a dispersed distribution and likely reflect false positives (discussed further below) from sequencing and/or bioinformatic errors (e.g. barcode collision) or rare mitotic recombination events. At the well-known recombination hotspot region *Esrrg1* (chr1:188,078,656–188,081,229, mm10)^8,28^ ReMIX identified 33 recombinant molecules in the sperm sample (Fig. 2b), while no recombinant molecules were identified in the corresponding genomic region in the somatic sample (Fig. 2c). Compared with previous studies involving more than 1500 mouse offspring, our results indicate that ReMIX is a powerful method for reconstruction of the fine-scale recombination landscape using gametes from a single individual.

We used both the number of recombinant molecules detected in the somatic sample and simulations (described in detail in below) to obtain independent estimates of the false-positive rate. Adjusting ReMIX results according to these estimations, our data suggest the total number of true crossovers along chromosome 1 in 1700 sperm to be 1540, giving an average of 0.9059 crossovers per meiotic product.

This corresponds well to the sex-averaged genetic map length of mouse chromosome 1 (90.9 cM), but is 8.3 cM longer than the map length of hybrid C57BL/6J × CAST/EiJ and hybrid CAST/EiJ × C57BL/6J males: 81 and 83.65, respectively, calculated from Table S1 of Paigen et al.^8^. This slightly higher number of observed recombinant molecules than expected based on the hybrid male map may have a biological basis (e.g. inter-individual variation^9^, inter-strain variation C57BL/6J vs. C57BL/6Ncrl^9,29^, and possible differences arising from quantification of recombination from viable offspring vs. quantification of recombination from gametes) or alternatively stem from detection errors (e.g. false negatives in the Paigen study^8^ due to lack of markers in the telomeric regions).

Finally, it has previously been shown that the genomic recombination landscape in mouse is positively correlated with CpG island density^30^. Here, we also find that recombinant molecules recovered by ReMIX are significantly closer to CpG islands than expected by chance based on 1000 permutations (Wilcox rank sum test, *p* < 2.5 × 10^−20^).

### Fine-scale recombination landscape in stickleback fish

We next evaluated the performance of ReMIX in an organism that has a recombination landscape with hotspots less intense than mouse. The threespine stickleback fish is an evolutionary genomics model organism with reasonably high-quality genome assembly, for which the recombination landscape has been previously described^31–33^. To match the mouse sample, we created gametic and somatic linked-read libraries each using 0.8 ng of HMW DNA (approximately equivalent to 1700 gametes) from sperm and kidney tissue of a freshwater Scottish stickleback strain (River Tyne).

The libraries were selected for a mean insert size of 600 bp and sequenced at 170× coverage on an Illumina HiSeq3000 machine. Similar to the mouse sample, the expected read coverage per molecule is ~0.1×. Both sets of linked-reads were analyzed using ReMIX and the stickleback reference genome (BROAD S1^34^, split into assembled scaffolds). 178M reads were retained post filtering and reconstructed into 21M molecules (eight linked-reads per molecule in average) of which 2639 (0.01%) were identified as recombinant by ReMIX.

**Fig. 3:**
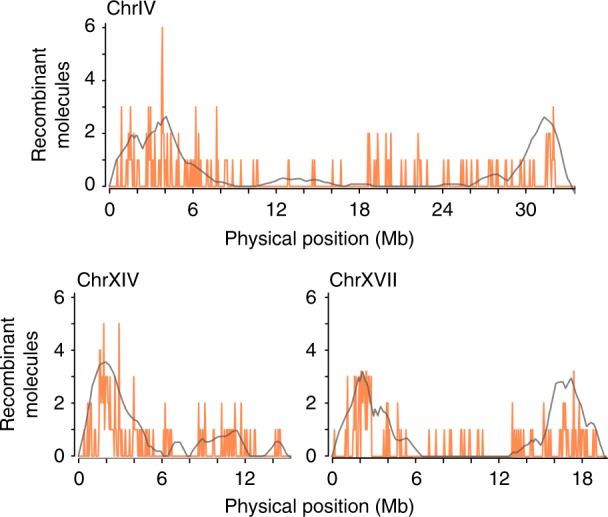
ReMIX recombination maps of example autosomes in a male freshwater stickleback. ReMIX analysis of linked-read data is plotted as the number of crossovers in 50 kb windows (orange). For comparison, recombination rate estimates obtained from a F2 lab cross^31^ of 140 males and 142 females individuals genotyped at 1872 markers are shown as gray line

The stickleback recombination landscape recovered with ReMIX follows the rate inferred from the previous low-resolution genetic map^31^ (Fig. 3 and Supplementary Fig. 7). Consistent with previous studies, ReMIX reveals recombination crossovers are enriched towards the distal ends of chromosomes and are significantly clustered compared to random expectations (Wilcox rank sum test *p* < 1 × 10^−20^). Similar to the mouse results, ReMIX recovered a number of recombination molecules in the stickleback somatic sample providing an indication of a modest false-positive rate (Supplementary Fig. 8). For most chromosomes the maximum number of these false-positive somatic recombinant molecules in 50 kb windows is 2 and we note some heterogeneity in the false-positive rates across chromosomes with elevated levels on chromosomes XIV, XIX, and XXI (as high as four molecules on chrXXI), which co-localize with scaffold ends and are likely scaffold assembly errors.

### Recombination suppression in inversion heterokaryotypes

When populations adapt to divergent environments in the face of ongoing gene flow, structural rearrangements, such as inversions have the potential to play an important role facilitating and maintaining adaptive divergence. By suppressing recombination in heterozygous individuals, inversions reduce the homogenizing effects of recombination in the local genomic region, allow the maintenance of linkage among neighboring mutations and the further accumulation of genetic differences between populations^17,35^. They therefore have the potential to act as adaptive cassettes if they harbor and maintain linkage among multiple beneficial mutations.

The recombination suppressing effects of inversions in heterozygotes is a well-known phenomena^36–38^ mediated in part by the abnormal formation of acentric and dicentric meiotic products due to improper resolution of double strand DNA breaks within inversion loops. The effects of an inversion on recombination can be heterogeneous—varying considerably along the affected chromosome^39^ with strong suppression around inversion breakpoints, partial suppression in the center of large inversions, and increased recombination in the genomic regions flanking the inversion and even on other chromosomes^38^.

Empirical quantification at the individual level of the strength and nature of recombination suppression around inversions requires analysis of a large number of meiotic products from focal individuals. Ideally, for testing the prevailing theory on the role of inversions in local adaptation, recombination should be studied in both alternative ecotypic (collinear) forms known to be undergoing adaptive divergence, as well as hybrids that are inversion heterokaryotypes. By overcoming the challenges related to expense and effort of previous methods, our ReMIX method enables us to investigate recombination variation within and among individuals and species on a scale that would previously have been difficult. A previous study^34^ identified three large inversions in the stickleback genome that show consistent orientation differences among multiple independent marine and freshwater populations. We focused on >5 Mb window centered one large 1.7 Mb inversion on chromosome XXI containing 76 genes and asked how the structural rearrangement influences the fine-scale recombination landscape within and among individuals, stickleback ecotypes, and their F1 hybrids. Linked-read genomic libraries were prepared from the DNA of ~3400 sperm from each of three marine and freshwater individuals from the Little Campbell River, Canada, and four F1 hybrids. Libraries were prepared and sequenced at the same coverage as described above and ReMIX analysis performed with the parameters fine-tuned for the genomic region and applied to all 10 individuals.

**Fig. 4:**
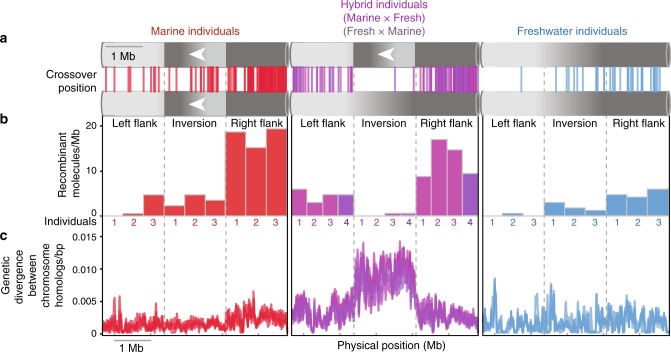
ReMIX analysis reveals recombination suppression in individuals heterozygous for a chromosomal inversion. **a**, **b** A 1.7 Mb inversion on stickleback chromosome XXI differs in orientation between marine (red) and freshwater (blue) ecotypes. Meiotic crossovers, detected by ReMIX as recombinant molecules, occur throughout the inversion in individuals homozygous for either orientation (three marine and freshwater fish, respectively). In contrast, inversion heterokaryotypes (heterozygous for inversion orientation, hybrids, *N* = 4, purple) show recombination suppression within the inversion and elevated rates of recombination in the regions flanking the inversion. **c** This recombination suppression allows the accumulation of linked genetic differences between the inverted haplotypes (shown for each individual as the number of heterozygous sites/bp in 20 kb windows)

We observed patterns of strong recombination suppression in F1 hybrids compared to their marine and freshwater homozygous counterparts (Fig. 4). In contrast to the numerous crossover events that were detected within the inversion in marine and freshwater inversion homozygotes, only two recombinant molecules were detected within the inversion among the ~13,600 gametes analyzed across four F1 hybrids (an effective recombination rate < 15% of inversion homozygotes). This pattern differs in the left and right inversion flanks where recombination appears to be comparatively high in hybrids exceeding the recombination rate observed in freshwater ecotypes. Finally, we observed considerable genetic divergence between the inversion orientations indicating recombination suppression substantially reduces the homogenizing effects of gene flow between the diverging ecotypes. The alternate orientations of this inversion therefore have the potential to harbor multiple, linked, beneficial mutations conferring an adaptive advantage to marine and freshwater ecotypes in the wild.

### ReMIX detects crossovers with high genomic resolution

**Fig. 5:**
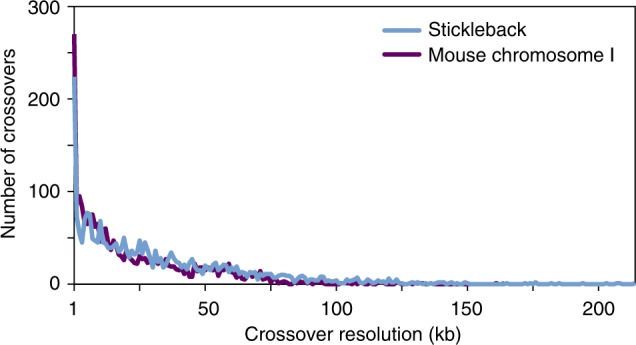
ReMIX detects recombination crossovers with high resolution in both mouse (purple) and stickleback (blue). After stringent filtering of reads ReMIX achieved a mean of 8.3 and 8.5 reads per molecule, and a median crossover resolution of 14 and 23 kb for mouse chromosome 1 and stickleback whole genome, respectively. This is considerably higher than previous studies of mice (e.g. median resolution of 225 kb in Paigen et al.^8^) and close to the maximally achievable resolution based on the biological constraint of distance between heterozygous sites in these strains. The highest crossover resolution we achieved was 1 bp in both mouse and sticklebacks, while only 1.22% and 4% of the crossovers detected had resolution as low as 100 kb or more for mouse and stickleback, respectively. We note that if desired, further improvements to crossover resolution up to the biological limit of distances between heterozygous sites could be achieved by increasing the depth of sequencing coverage (and consequently the number of reads per molecule)

A recombinant molecule is composed of two continuous sections: *s*_*a*_ phased to one haplotype and *s*_*b*_ phased to the opposite haplotype. The crossover may have occurred anywhere between the last informative variant of *s*_*a*_ and the first informative variant of *s*_*b*_. Thus, we consider the resolution of a crossover as the physical distance between these two informative variants. By taking advantage of long-range molecular data spanning high-quality heterozygous variants segregating within a single individual, ReMIX directly identifies the recombinant molecules with high accuracy and crossover resolution (Fig. 5). The achievable crossover resolution of our approach is limited primarily by the density of heterozygous sites within an individual (something that varies considerably across taxa), and secondarily by the sequencing coverage used to detect these informative sites. For example, based on whole genome-sequencing data, we estimate hybrid C57BL/6Ncrl × CAST/EiJ mouse and freshwater stickleback individuals used in this study will have a median distance of 44 and 63 bp between heterozygous sites, respectively.

### Analysis of accuracy on simulated data

Since fine-scale recombination rate can vary considerably among individuals of the same species, comparisons of our ReMIX results with previously published recombination studies provides only a qualitative assessment of the accuracy of our pipeline. To achieve a better indication of ReMIX’s performance, we simulated several data sets using the linked-read simulator LRSIM^40^. Starting from a reference sequence as an input, LRSIM can simulate diploid sequences with a user-specified number of heterozygous SNPs, indels and structural variants. Then the simulator extracts paired-end reads from each haplotype and assigns the reads to molecules by attaching the specific 10× barcodes depending on a user-specified number of reads per molecule. In order to validate our method, we generated linked-read sets containing both non-recombinant and recombinant molecules. To achieve this, we first used LRSIM to create a set of linked-reads containing only non-recombinant molecules. Then we simulated crossovers between the two haplotypes (a switch of haplotype state) generated by LRSIM in the first run and we ran LRSIM on the recombinant haplotypes to obtain a second set of linked-reads containing recombinant molecules (those spanning the simulated crossovers). The resulting molecule sets were merged to simulate the mix of recombinant and non-recombinant molecules present in a pool of gametes. The sensitivity (or the true positive rate) is then computed as the proportion of the recombinant molecules correctly identified by ReMIX out of the total set of simulated recombinant molecules.

**Fig. 6:**
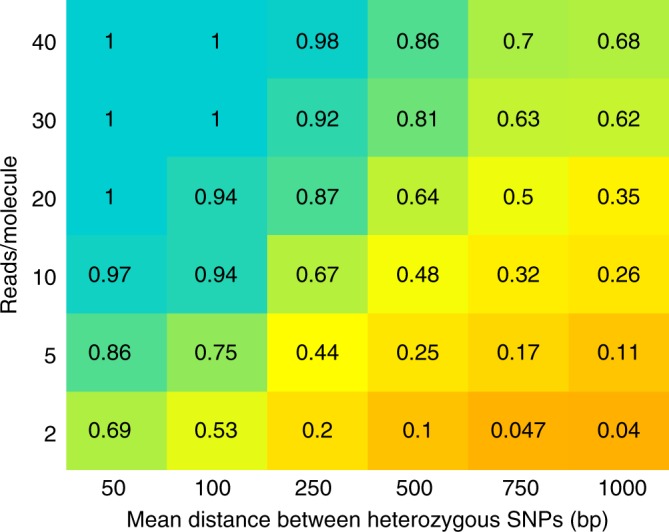
ReMIX detects recombinant molecules with high sensitivity. In simulated data, the sensitivity of ReMIX to detect recombinant molecules (low = orange; high = blue; and numbers within boxes) is high at moderate to high heterozygosity levels (*x*-axis) and moderate to high sequencing depth (*y*-axis)

Let *m* be a recombinant molecule with two contiguous segments *s*_*a*_ and *s*_*b*_ phased to opposite haplotypes. ReMIX is able to detect *m* only if reads from both *s*_*a*_ and *s*_*b*_ are spanning heterozygous variants. Thus, the heterozygosity of the organism and the sequencing coverage are two parameters that influence the sensitivity of ReMIX to detect true positive recombinants. To evaluate the sensitivity, we performed simulations with different heterozygosity levels and read density per molecule. The positions of heterozygous SNPs and reads were chosen randomly for each run. For each parameter configuration we ran the simulations 10 times and averaged the sensitivity values. We show that ReMIX is highly sensitive (with more than 90% of recombinant molecules detected at moderate to high levels of heterozygosity and moderate to high sequencing depth (Fig. 6).

The percentage of correctly reported molecules slowly decreases with the increase of the distance between the heterozygous variants. This is caused by the lower probability of reads spanning informative variants flanking recombination crossovers when an organism has a lower level of heterozygosity. However, ReMIX sensitivity can be easily increased in those cases by using a higher sequencing coverage.

Similar to other pipelines constructed for processing linked-reads^26,41^, the performance of ReMIX is dependent on the reaction conditions during the 10X Genomics linked-read library generation. One major consideration is the probability of two independent molecules from the same locus in the genome being assigned the same barcode (barcode collision). This depends on the amount of DNA in the reaction (which influences the number of molecules per droplet (GEM)), and the genome size of the organism in question. When preparing libraries from the same weight of DNA, small genomes will have a higher molecular copy number of each genomic locus, compared to large genomes. This leads to a higher probability of barcode collision of molecules from the same genomic locus due to a higher probability of them being trapped within the same GEM. In organisms with small genomes, using less DNA in the linked-read library preparation reaction can mitigate the occurrence of barcode collision.

If barcode collision occurs among alternate haplotypes, this has the potential to lead ReMIX to identify false-positive recombinant molecules. Let *m*_1_ and *m*_2_ be two molecules of opposite haplotype state, from the same genomic region, that have the same barcode. The short reads are regrouped into molecules based on their barcode and a parameter specifying the maximal genomic distance separating two reads of the same molecule. Depending on the *m*_1_ and *m*_2_ read positions, the two molecules are detected as one molecule with two contiguous segments phased to opposite haplotypes. As a consequence, ReMIX reports the merged molecules as a recombinant molecule. Finally, identification of false-positive recombinant molecules might also be caused by erroneous read mapping, structural variants, and reference genome assembly errors.

To address these two different causes of false positives, we used the complete mouse reference genome (mm10) to simulate a close to real case scenario for the numbers of molecules per GEM and the density and length of structural variants. Using the method described above, we simulated seven molecules per GEM, the mean number of molecules per GEM that we obtained with our empirical mouse and stickleback data sets, and also 10 molecules per GEM, the maximum number reported by 10X Genomics. We then ran ReMIX on both sets and grouped the reported molecules in 100 kb windows (Table 1). Under conditions matching our empirical datasets we estimated a low recombinant molecule false-positive rate with a large majority of the intervals not containing any false-positive molecules (94.9%) and only 5% of intervals showing false positives. This increased to 10.37% of intervals when the number of molecules per GEM was simulated to be 10.

**Table 1:** Number of false positive (FP) molecules identified by ReMIX at 7 and 10 molecules per GEM

No. of FP per 100 kb window	0	1	2	3	Total ratio of windows with FP (%)
7 mol/GEM	25,889	1335	44	1	5.06
10 mol/GEM	24,440	2678	145	6	10.37

After splitting the mouse genome in 100 kb windows (total of 27,269), we report the number of FP molecules identified by ReMIX in each window

The distribution of the intervals containing false-positive molecules for mouse chromosome 1 for 7 and 10 molecules per GEM is shown in Supplementary Figs. 9 and  10, respectively. Similar levels of false positives were detected on the other mouse chromosomes. We note that due to the stringent filters of our pipeline (see the “Methods” section), structural variants were filtered, and did not have an impact on the false-positive rate. Since the false-positive molecules are uniformly distributed across the genome and do not cluster in specific regions, they do not interfere with the detection of regions with high recombination activity. And for organisms with low recombination rate the false positives detected by ReMIX can be decreased by lowering the amount of DNA used in the library preparation reaction, and the use of multiple independent reactions. This will decrease the mean number of molecules per GEM while maintaining the number of total recombinant molecules captured from the gametes.

## Discussion

Understanding the extent and molecular basis of recombination variation has been challenging due to the expense of creating individualized high-resolution genome-wide recombination maps. Here we present a cost and time effective method to build individualized recombination maps from pooled gamete DNA. This method makes use of linked-read sequencing technology developed by 10X Genomics to acquire long-range haplotype information from gametes of a single individual. Our specialized bioinformatics pipeline named ReMIX then faithfully identifies recombinant molecules from the linked-read data produced. Using these recombinant molecules, crossover locations are defined as genomic intervals based on the location of the last variant of the first haplotype and first variant of the second. We demonstrate the application of our method by building fine-scale recombination maps for a male mouse, an organism with well-characterized recombination hotspots, and a less traditional model organism, a male threespine stickleback fish.

We validated our method through comparisons to previously reported recombination landscapes in mouse^8^ and sticklebacks^31^, and simulations to quantify sensitivity and specificity. Our approach faithfully identified known recombination hotspots on mouse chromosome 1 with high resolution (median of 14 kb), and revealed enrichment in crossovers at the distal end of autosomes in the male mouse, and both ends of chromosomes in the male stickleback. Through simulations we show ReMIX has high sensitivity and that for organisms with low levels of heterozygosity this sensitivity can be increased by sequencing the linked-read library to higher coverage. In addition, we used DNA extracted from somatic tissue as a control to test the specificity of our method. The use of a somatic control enabled the estimation of background noise in the data set that might be caused by bioinformatic error, reference genome assembly errors, copy number and structural variants, or rare mitotic recombination. Our results show that the true meiotic recombination signal stands out amidst the more dispersed noise from false positives, indicating ReMIX to be a reliable approach for constructing and studying variation in fine-scale recombination landscapes. Individualized genome-wide recombination maps that were previously constructed from extensive genotyping in thousands of offspring or whole genome sequencing of individual gametes^25^ can now be produced with less time and effort by applying our novel method to pools of gametes from a single individual.

The whole genome recombination landscape we obtained for a male C57BL/6Ncrl × CAST/EiJ mouse (Supplementary Fig. 5) is in agreement with the reported observation that male recombination activity is concentrated at the distal end of the autosomes. We also detected previously reported mouse chromosome 1 hotspots (*Esrrg1* and *Hlx1* (Fig. 2b and Supplementary Fig. 3b) in our data set. Using a sliding window approach by counting number of haplotypes switching molecules per 5 kb interval, we find a 9 kb interval at chr1:188,079,000–188,088,000 (mm10) region with highest recombination activity. This region spans the known *Esrrg1* hotspot. Crossovers were identified from 31 haplotype switching molecules out of 1736 total mapped molecules in that 9 kb interval, suggesting a recombination rate of 1.78 cM in 9 kb. PRDM9, a protein with histone methyltransferase activity, plays an important role in recombination hotspots in many mammals including mice and humans. Consistent with previous studies showing that recombination in this region is mediated by PRDM9^28^, we find the PRDM9 motif specific to *Esrrg1* located within the 9 kb hotspot (Fig. 2). Similarly, following the criteria used by Liu et al.^42^, we also detect extended genomic regions (≥500 kb) without any recombination crossovers (putative cold spots). The 167 regions detected span a total of 194 Mb (~7% of the mouse genome, similar to the proportion reported by Liu et al.^42^) and include well-characterized coldspot on chr12 at ~20 Mb that has been reported in multiple different strains^43^.

Mouse and stickleback recombination crossovers are not distributed randomly across the genome, but are rather significantly clustered and more proximal to CpG islands than expected by chance. In stickleback, the region with the highest recombination activity is located on chromosome IV at ~3.8 Mb. Here, within a 7 kb interval, we detected six recombinant molecules out of a total of 1366 mapped molecules. This corresponds to a recombination rate of 0.44 cM, roughly one quarter the intensity of the mouse hotspot described above.

Megabase-sized genomic inversions are predicted to facilitate divergent adaptation and speciation with ongoing gene flow. Through empirical evaluation of thousands of gametes from multiple marine, freshwater, and hybrid individuals, we have shown that a large chromosomal inversion on stickleback chrXXI causes strong recombination suppression within inversion heterokaryotypes, elevated recombination in the immediate flanking regions and harbors a high density of linked-mutations. While many studies have shown strong recombination suppression effects of inversions between species our study illustrates how recombination modifiers, such as inversions can cause strong recombination suppression even between adaptively diverging populations in the early stages of speciation.

We have demonstrated our method here using DNA extracted from sperm in organisms with high-quality genome assemblies. Considering the ease of collecting pools of gametes, and the low amount of input DNA required (e.g. 1 ng for a genome size of 3 GB genome, or <1 ng for smaller genomes), we anticipate our method can be extended to a wide range of organisms. ReMIX can detect recombination events in parts of the genome with diploid chromosome homologs that have heterozygous markers. Therefore, individualized recombination maps can be constructed for the whole genome including recombining regions of sex chromosome in the homogametic sex and pseudoautosomal regions of sex chromosomes in the heterogametic sex. While not shown here, the same principle could be expanded to study recombination in polyploids.

Our approach allows empirical quantification of fine-scale variation in recombination of both model and non-model organisms, including individuals sampled from the wild. We highlight that, for organisms whose genome assembly is lacking or of low quality, a de novo diploid assembly can be built^41^ using the same linked-read data set generated from gametes. This de novo assembly can then be used as the reference genome for ReMIX analysis of recombination. By overcoming the challenges related to expense and effort of previous methods, our ReMIX pipeline, opens up numerous possibilities for investigating recombination variation within and among individuals, including the exciting potential of using forward genetic mapping to dissect and identify the molecular basis of recombination variation.

## Methods

### Extracting HMW genomic DNA

Stickleback genomic DNA was isolated from kidneys and sperm of a male wild-derived freshwater fish (River Tyne, Scotland). The sperm were collected via testes maceration in Hank’s solution and purified to remove any potential contaminating diploid cells using a Nidacon PureSperm^®^ gradient following the manufacturer’s instructions with slight modifications. PureSperm gradient was made with 40/60/90 percentage solution and centrifuged at 300 × *g* for 30 min. Purified sperm cells were resuspended in 1×PBS (ThermoFisher, Cat. no. 10010023). Kidneys from the same male fish were dissected and rinsed in PBS prior to DNA extraction. HMW gDNA was extracted from purified sperm cells and kidney using Qiagen Magattract HMW DNA extraction kit (Cat. no. 67563) following the protocol outlined in 10X Genomics Chromium Genome User Guide Rev B^44^. We followed the Genomic DNA extraction from cell suspension protocol for the sperm sample, and the Tissue DNA extraction protocol for the kidney sample.

Mouse genomic DNA was isolated from F1 hybrid (C57BL/6Ncrl × CAST/EiJ) male spleen and sperm cells. Sperm were collected from the cauda epididymis of a 7-week-old F1 male hybrid mouse following Ijiri et al.^45^. Extracted epididymis were finely chopped in 1 × PBS. After settling for 3–5 min at room temperature, the supernatant containing viable sperm was purified by gradient centrifugation at 300 × *g* for 20 min at room temperature (PureSperm 40/80; Nidacon International, Goteborg, Sweden). For somatic DNA control, excised spleen tissue was crushed between frosted glass microscope slides to make single cell suspension. Purified sperm and spleen cells were subsequently used for the isolation of HMW genomic DNA following Wu et al.^46^.

The quality of extracted HMW DNA was checked by pulse field gel electrophoresis. All gametic and somatic samples showed a gradient of HMW DNA > 50 kb in size. This corresponds well to the described conditions for optimal performance of 10X Genomics linked-read library preparation^44^.

### Constructing linked-read sequencing libraries

We used a Chromium controller instrument (10X Genomics^®^) to partition input DNA into nanoliter-sized droplets and prepare linked-read libraries following the manufacturer’s instructions (10X Genomics Chromium Controller User Manual) for input DNA quantification, dilution, GEM generation, and library preparation. For stickleback, we used ~0.8 ng of HMW genomic DNA as input (equivalent to ~1700 haploid genomes). To achieve the equivalent number of haploid genomes for mouse (1700), we carried out six parallel reactions with 1.2 ng input DNA for each of the sperm and somatic samples. In the Chromium Controller, input DNA was partitioned into ~1 million droplets (GEMs), each containing reagents with a unique barcode (Gemcode). The droplets were recovered from the microfluidic chip and isothermally incubated (at 30 °C) for ~3 h to produce barcoded short reads, average size ~700 bp, from each template DNA within each droplet. Following the isothermal incubation, the post GEM reads were recovered, then purified and size selected using Silane and Solid phase reverse immobilization (SPRI) beads. Illumina-compatible paired-end sequencing libraries were then prepared following 10X Genomics instructions, with 10 cycles of PCR. The final library comprises reads with a standard Illumina P5 adapter, followed by a 16 bp 10X Genomics barcode at the start of read 1, the genomic DNA insert, and an 8 bp sample index at the P7 adapter end. The final library was size selected to an average size of 600 bp. Sequencing was conducted with an Illumina HiSeq 3000 instrument with 2 × 150 bp paired-end reads. Each library was sequenced to ~170× genome coverage. This is equivalent to ~0.1× read coverage per molecule for the ~1700 haploid gametes in the input.

### ReMIX pipeline for identifying recombinant molecules

ReMIX pipeline contains three main steps: identifying high-quality heterozygous variants, reconstructing molecules, and haplotype phasing each molecule to determine the recombinant molecules and the position of their crossovers (Supplementary Fig. 1). We make use of the software provided by 10X Genomics for reference guided analysis of linked-read data (Long Ranger^47^), but deviate from it in many places. After testing multiple equivalent tools for read filtering, alignment, or variant calling, we have configured ReMIX with the combination of tools for which we obtained the best results using both simulated and real data.

### Identifying high-quality heterozygous variants

ReMIX’s detection of recombinant molecules is based on the estimation of the two haplotypes present in the diploid individual analyzed. The accuracy of this estimation depends on the quality and frequency of heterozygous variants identified by our pipeline. Thus, in the first step of ReMIX (Supplementary Fig. 1) we remove the linked-reads containing sequencing errors in their genomic sequence, align the correct linked-reads on a reference genome, call the set of variants, and apply a hard filter on this set.

In step 1 of ReMIX (Supplementary Fig. 1 Step 1), the linked-reads are extracted from the Illumina’s sequencer base call files (*.bcl) using *Long Ranger mkfastq*^47^, and then filtered and trimmed with *Cutadapt*^48^, *Trimmomatic*^49^, and *Long Ranger basic*^47^. The linked-reads with 16 bp barcode sequences matching the barcode whitelist provided by 10X Genomics are aligned with *bwa mem*^50^ to the reference genome. The duplicates are marked with *Picard tools*^51^ and read alignment around indels is improved using *GATK’s IndelRealigner*^52^. ReMIX identifies variants with *samtools mpileup*^53^ and applies a first variant filter using *bcftools*^54^ to extract high-quality heterozygous variants with low allelic bias. Specifically, we excluded variants with strand-, mapping-quality-, read-position- or base-quality bias, variants with extreme low or high depth of coverage, and variants with low genotype or variant quality scores using the following thresholds: Mann–Whitney *U*-test of mapping quality bias (MQB) < 0.4; Mann–Whitney *U*-test of base quality bias (BQB) < 0.4; Mann–Whitney *U*-test of of mapping quality vs. strand bias MQSB < 0.8; Mann–Whitney *U*-test of read position bias (RPB) < 0.4; Maximum fraction of reads supporting an indel IMF < 0.1 or IMF > 0.9; Approximate read depth DP < 5 or DP > 220; genotype quality (GQ) < 30; variant quality QUAL < 100.

### Reconstructing molecules

At the end of the first step of ReMIX the linked-reads are not yet organized into molecules. The purpose of the second step is to reconstruct the molecules, so that the haplotype phasing algorithm can take advantage of the long-range information available.

The linked-reads generated from the same DNA molecule carry identical barcodes. However, since multiple molecules (e.g. 10) from diverse locations in the genome are typically trapped within the same GEM droplet and tagged with the same barcode, the molecules cannot be reconstructed based only on the barcodes of the linked-reads. From the quality control steps following HMW DNA extraction, it is possible to obtain an estimate of the expected average size of HMW DNA molecules in the reaction. Thus, we can link reads sharing an identical barcode into the same molecule if they aligned to the neighborhood of a genomic region with total molecule span similar to the expected average molecule size.

Still, this process does not always prevent linkage of reads from two or more independent molecules into a single reconstructed molecule when the original molecules share the same barcode and originate from the same genomic region. We refer to this case as *barcode collision*. For linked-read libraries constructed from organisms with large genome size using a low amount of input DNA in the library generation process, the probability of a single GEM droplet containing two HMW DNA molecules from the same genomic region is small, but non-zero. For example, the probability of barcode collision is ~3.2 × 10^−3^ for linked-read libraries prepared from 1 ng of mouse DNA of 60 kb average molecular weight, given a total mouse genome size of 3 Gb (meaning ~3200 of 1M GEMs will contain more than one molecule from the same region of the genome). When the original molecules are generated from opposite haplotypes, the barcode collision cases can generate *recombinant-like* molecules that will be identified by ReMIX as false positive. To limit the number of false positives, we introduced the following parameters: the maximum molecule length, the maximum distance between two consecutive linked-reads grouped into the same molecule and the minimum and maximum number of expected linked-reads per molecule. The values of these parameters depend on the library construction and sequencing parameters.

For this second step of ReMIX we constructed a Long Ranger sub-pipeline called Long Ranger ReportMolecules (Supplementary Fig. 1 Step 2). This sub-pipeline is based on two parts of the Long Ranger Whole Genome Phasing and structural variant calling (SV Calling) pipeline (*Long Ranger wgs*)^47^: the computational reconstruction of the molecules, and the report of the molecule information in the INFO field of the variant call format (vcf) file. Long Ranger ReportMolecules incorporates a number of changes to the original Long Ranger pipeline including the parameters mentioned above: the maximum molecule length, the minimum and maximum number of expected linked-reads per molecule. The input of this sub-pipeline is the binary sequence alignment map (bam) file with high-quality-mapped reads including a tag with their respective barcodes, and the vcf file with the filtered heterozygous variants. Long Ranger ReportMolecules outputs a file that reports for each molecule: the genomic start and end position; the barcode; and the number of reads. This is accompanied by a modified vcf file that for each variant contains the reconstructed molecules spanning each of the alleles of this variant. Molecules with extreme low coverage (<6 reads) are excluded from further analysis.

### Haplotype phasing molecules

In the last step, ReMIX estimates the two haplotypes by phasing selected variants based on the molecule information previously obtained. Then, depending on the alleles spanned by the reads of a molecule, the molecule is considered as belonging to one of the two haplotypes or as being a recombinant molecule.

Structural variants such as deletions, duplications, copy number variations, or translocations can cause errors in the read alignment, and thus variants can be incorrectly called in these regions. The false variants then interfere with the phasing process and introduce errors in the estimated haplotypes. Moreover, the structural variants can generate *barcode collision*-like cases. If misplaced reads and a real molecule share the same barcode and are aligned in the same genomic region, the algorithm used for reconstructing the molecules regroups the misplaced reads and the real molecule in a unique molecule. When the misplaced reads and the real molecule originate from opposite haplotypes, the reconstructed molecule appears as if it would span a crossover event as presented in Supplementary Fig. 11. ReMIX identifies these problematic regions by removing: variants that have a notable difference between the molecular or read coverage compared to the mean values for their chromosome; and variants for which the read coverage is uneven between the alleles.

The remaining variants are then phased with HBOP^55^ based on the molecules computed during the second step. HBOP is a single individual phasing algorithm that can take into account reads belonging to a longer DNA fragment and therefore capitalizes on the long-range information of the molecule during phasing.

The two haplotypes constructed by HBOP are then used to phase each molecule. For each variant spanned by a molecule with at least one read, we consider the haplotype of the covered allele and the sequencing quality score at that position. Then, based on a score function implemented in Long Ranger wgs^47^, we compute for each molecule the probability of belonging to the two haplotypes or being a mix of the two. Contrary to Long Ranger wgs, we do not consider the molecules that contain reads spanning both alleles of a variant, since this behavior is likely to arise from a barcode collision. Once the probabilities are computed for each molecule, we filter again to remove variants showing an allelic bias in the number of molecules phased to each allele. Depending on the quality of the reference sequence used in the mapping process or on the copy number variation, some of the structural variants are still unidentified and can introduce errors in the process of determining the recombinant molecule. We then recompute the haplotype probabilities for each molecule.

From the set of molecules that have a high probability of belonging to a mixture of two haplotypes states, ReMIX considers as truly recombinant the molecules for which we can identify a clear crossover position: a minimum number of variants and a minimum ratio of variants phased to the same haplotype on each side of the crossover. We then output for each recombinant molecule the genomic start and end position; the crossover positions; the barcode; and the number of reads.

All animals used in this study were housed at approved animal facilities and handled according to Baden-Württemberg State approved protocols (Competent authority: Regierungspräsidium Tübingen, Germany; Permit and notice numbers 35/9185.82-5, 35/9185.46)

### Reporting summary

Further information on research design is available in the Nature Research Reporting Summary linked to this article.

## Supplementary information

Supplementary Information

Reporting Summary

Source Data

## Data Availability

The datasets generated and analyzed in the current study are available in the NCBI short read repository [https://www.ncbi.nlm.nih.gov/bioproject/PRJNA562078]. All other relevant data is available upon request.

## Code availability

ReMIX source code can be found at github [https://github.com/adreau/ReMIX] and zenodo archive 10.5281/zenodo.3351406.

## References

[1] Fledel-Alon A (2009). Broad-scale recombination patterns underlying proper disjunction in humans. PLoS Genet..

[2] Hassold T, Hunt P (2001). To err (meiotically) is human: the genesis of human aneuploidy. Nat. Rev. Genet..

[3] Inoue K, Lupski JR (2002). Molecular mechanisms for genomic disorders. Annu. Rev. Genomics Hum. Genet..

[4] Wang S, Zickler D, Kleckner N, Zhang L (2015). Meiotic crossover patterns: obligatory crossover, interference and homeostasis in a single process. Cell Cycle.

[5] Coop G, Wen XQ, Ober C, Pritchard JK, Przeworski M (2008). High-resolution mapping of crossovers reveals extensive variation in fine-scale recombination patterns among humans. Science.

[6] Ptak SE (2005). Fine-scale recombination patterns differ between chimpanzees and humans. Nat. Genet..

[7] Kong A (2010). Fine-scale recombination rate differences between sexes, populations and individuals. Nature.

[8] Paigen K (2008). The recombinational anatomy of a mouse chromosome. PLoS Genet..

[9] Koehler KE, Cherry JP, Lynn A, Hunt PA, Hassold TJ (2002). Genetic control of mammalian meiotic recombination. I. Variation in exchange frequencies among males from inbred mouse strains. Genetics.

[10] Nachman MW, Payseur BA (2012). Recombination rate variation and speciation: theoretical predictions and empirical results from rabbits and mice. Philos. Trans. R. Soc. B.

[11] Comeron JM, Ratnappan R, Bailin S (2012). The many landscapes of recombination in *Drosophila melanogaster*. PLoS Genet..

[12] Dumont BL, White MA, Steffy B, Wiltshire T, Payseur BA (2011). Extensive recombination rate variation in the house mouse species complex inferred from genetic linkage maps. Genome Res..

[13] Ma J (2010). Recombinational landscape of porcine X chromosome and individual variation in female meiotic recombination associated with haplotypes of Chinese pigs. BMC Genomics.

[14] Fernandez AI (2014). Recombination of the porcine X chromosome: a high-density linkage map. BMC Genet..

[15] Hill WG, Robertson A (1966). The effect of linkage on limits to artificial selection. Genet. Res..

[16] Felsenstein J (1974). The evolutionary advantage of recombination. Genetics.

[17] Kirkpatrick M, Barton N (2006). Chromosome inversions, local adaptation and speciation. Genetics.

[18] Li HH (1988). Amplification and analysis of DNA sequences in single human sperm and diploid cells. Nature.

[19] Carrington M, Cullen M (2004). Justified chauvinism: advances in defining meiotic recombination through sperm typing. Trends Genet..

[20] Kauppi L, Jeffreys AJ, Keeney S (2004). Where the crossovers are: recombination distributions in mammals. Nat. Rev. Genet..

[21] Broman KW, Murray JC, Sheffield VC, White RL, Weber JL (1998). Comprehensive human genetic maps: individual and sex-specific variation in recombination. Am. J. Hum. Genet..

[22] Kong A (2002). A high-resolution recombination map of the human genome. Nat. Genet..

[23] Shifman S (2006). A high-resolution single nucleotide polymorphism genetic map of the mouse genome. PLoS Biol..

[24] Smeds L, Mugal CF, Qvarnstrom A, Ellegren H (2016). High-resolution mapping of crossover and non-crossover recombination events by whole-genome re-sequencing of an avian pedigree. PLoS Genet..

[25] Wang JB, Fan HC, Behr B, Quake SR (2012). Genome-wide single-cell analysis of recombination activity and de novo mutation rates in human sperm. Cell.

[26] Zheng GXY (2016). Haplotyping germline and cancer genomes with high-throughput linked-read sequencing. Nat. Biotechnol..

[27] Cox A (2009). A new standard genetic map for the laboratory mouse. Genetics.

[28] Billings T (2013). DNA binding specificities of the long zinc-finger recombination protein PRDM9. Genome Biol..

[29] Fontaine DA, Davis DB (2016). Attention to background strain is essential for metabolic research: C57BL/6 and the international knockout mouse consortium. Diabetes.

[30] Han L, Su B, Li WH, Zhao ZM (2008). CpG island density and its correlations with genomic features in mammalian genomes. Genome Biol..

[31] Roesti M, Moser D, Berner D (2013). Recombination in the threespine stickleback genome—patterns and consequences. Mol. Ecol..

[32] Glazer AM, Killingbeck EE, Mitros T, Rokhsar DS, Miller CT (2015). Genome assembly improvement and mapping convergently evolved skeletal traits in sticklebacks with genotyping-by-sequencing. G3-Genes Genom. Genet..

[33] Sardell JM (2018). Sex differences in recombination in sticklebacks. G3-Genes Genom. Genet..

[34] Jones FC (2012). The genomic basis of adaptive evolution in threespine sticklebacks. Nature.

[35] Charlesworth B, Barton NH (2018). The spread of an inversion with migration and selection. Genetics.

[36] Dobzhansky T, Sturtevant AH (1938). Inversions in the chromosomes of *Drosophila pseudoobscura*. Genetics.

[37] Novitski E, Braver G (1954). An analysis of crossing over within a heterozygous inversion in *Drosophila melanogaster*. Genetics.

[38] Stevison LS, Hoehn KB, Noor MAF (2011). Effects of inversions on within- and between-species recombination and divergence. Genome Biol. Evol..

[39] Sturtevant, A. H. & Dobzhansky, T. *Contributions to the Genetics of Certain Chromosome Anomalies in Drosophila melanogaster*. (Carnegie Institution of Washington, 1931).

[40] Luo RB, Sedlazeck FJ, Darby CA, Kelly SM, Schatz MC (2017). LRSim: a linked-reads simulator generating insights for better genome partitioning. Comput Struct. Biotec.

[41] Weisenfeld NI, Kumar V, Shah P, Church DM, Jaffe DB (2017). Direct determination of diploid genome sequences. Genome Res..

[42] Liu EY (2014). High-resolution sex-specific linkage maps of the mouse reveal polarized distribution of crossovers in male germline. Genetics.

[43] Morgan AP (2017). Structural variation shapes the landscape of recombination in mouse. Genetics.

[44] 10x Genomics, Chromium™ Genome Reagent Kit User Guide (2018).

[45] Ijiri TW, Merdiushev T, Cao W, Gerton GL (2011). Identification and validation of mouse sperm proteins correlated with epididymal maturation. Proteomics.

[46] Wu Q, Chen M, Buchwald M, Phillips RA (1995). A simple, rapid method for isolation of high-quality genomic DNA from animal tissues. Nucleic Acids Res..

[47] Long Ranger. 10X Genomics. https://support.10xgenomics.com/genome-exome/software/pipelines/latest/what-is-long-ranger (2018).

[48] Martin M (2011). Cutadapt removes adapter sequences from high-throughput sequencing reads. EMBnet. J..

[49] Bolger AM, Lohse M, Usadel B (2014). Trimmomatic: a flexible trimmer for Illumina sequence data. Bioinformatics.

[50] Li H, Durbin R (2009). Fast and accurate short read alignment with Burrows–Wheeler transform. Bioinformatics.

[51] Picard toolkit. Broad Institute, GitHub repository. http://broadinstitute.github.io/picard/ (2018).

[52] McKenna A (2010). The Genome Analysis Toolkit: a MapReduce framework for analyzing next-generation DNA sequencing data. Genome Res..

[53] Li H (2011). A statistical framework for SNP calling, mutation discovery, association mapping and population genetical parameter estimation from sequencing data. Bioinformatics.

[54] Li H (2009). The sequence alignment/map format and SAMtools. Bioinformatics.

[55] Xie M, Wang J, Jiang T (2012). A fast and accurate algorithm for single individual haplotyping. BMC Syst. Biol..

