# Supplementary material for: Genome-wide recombination map construction from single individuals using linked-read sequencing
Source: Nat Commun. 2019 Sep 20;10:4309. doi: 10.1038/s41467-019-12210-9 (PMC6754380; doi:10.1038/s41467-019-12210-9)
Supplement: Supplementary file 1 — Supplementary Information [file 41467_2019_12210_MOESM1_ESM.pdf]

Supplementary information for

Genome-wide recombination map construction from single individuals using linked-read sequencing

Andreea Dréau[1], Vrinda Venu[1], Elena Avdievich[1], Ludmila Gaspar[1], Felicity C. Jones[1, *]

[1] Friedrich Miescher Laboratory of the Max Planck Society, Max-Planck-Ring 9, 72076 Tübingen, Germany

*Correspondence:

Dr Felicity C. Jones
Jones Lab
Friedrich Miescher Laboratory of the Max Planck Society
Max-Planck-Ring 9
72076 Tübingen
Germany
fcjones@tuebingen.mpg.de
Ph: +49(0)7071 601 840

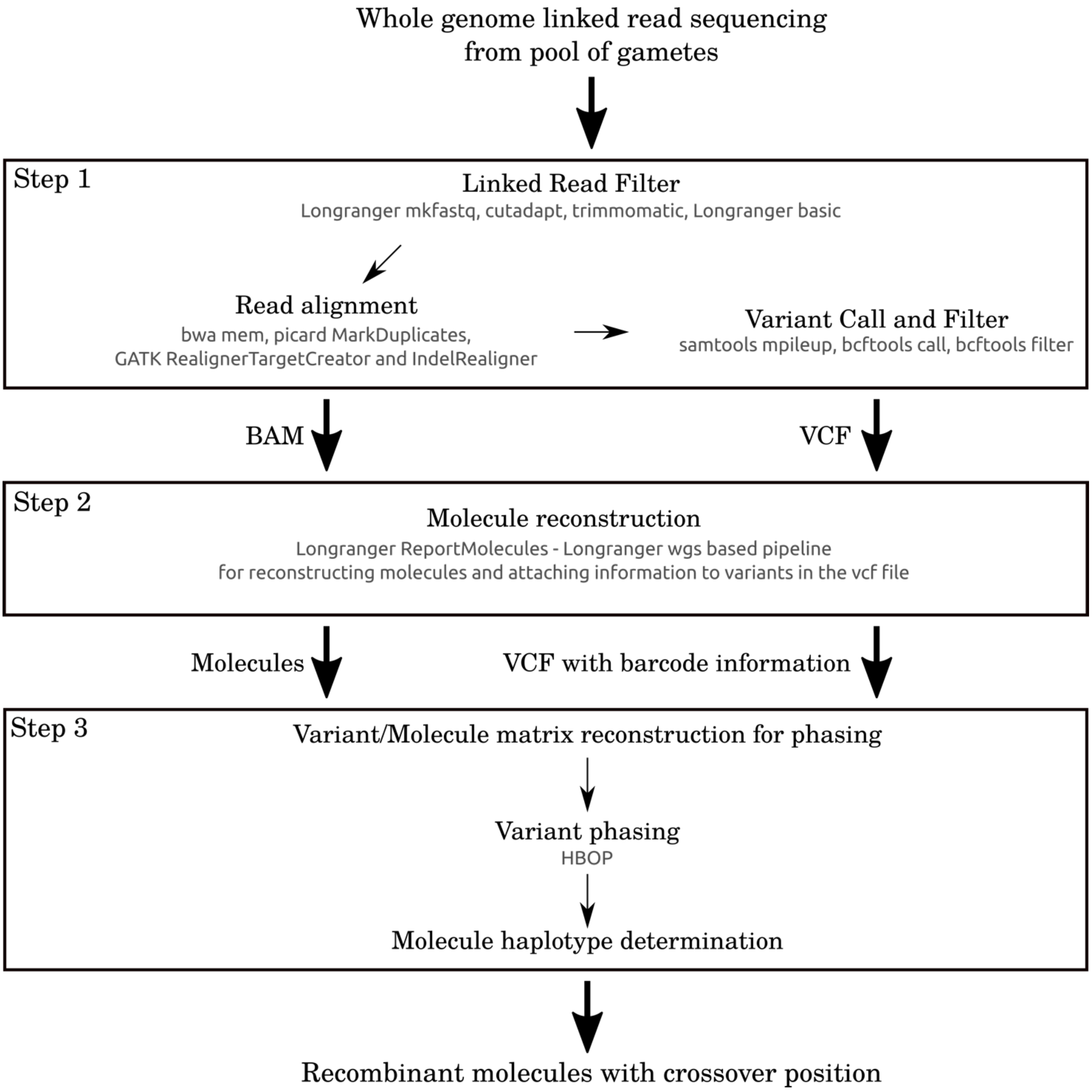

Supplementary Figure 1: ReMIX pipeline's main steps: identification of high-quality heterozygous variants, reconstruction of molecules, and haplotype phasing each molecule. In the input our pipeline requires Illumina's base call files from sequencing pool of gametes and outputs the identified recombinant molecules and the position of their crossovers.

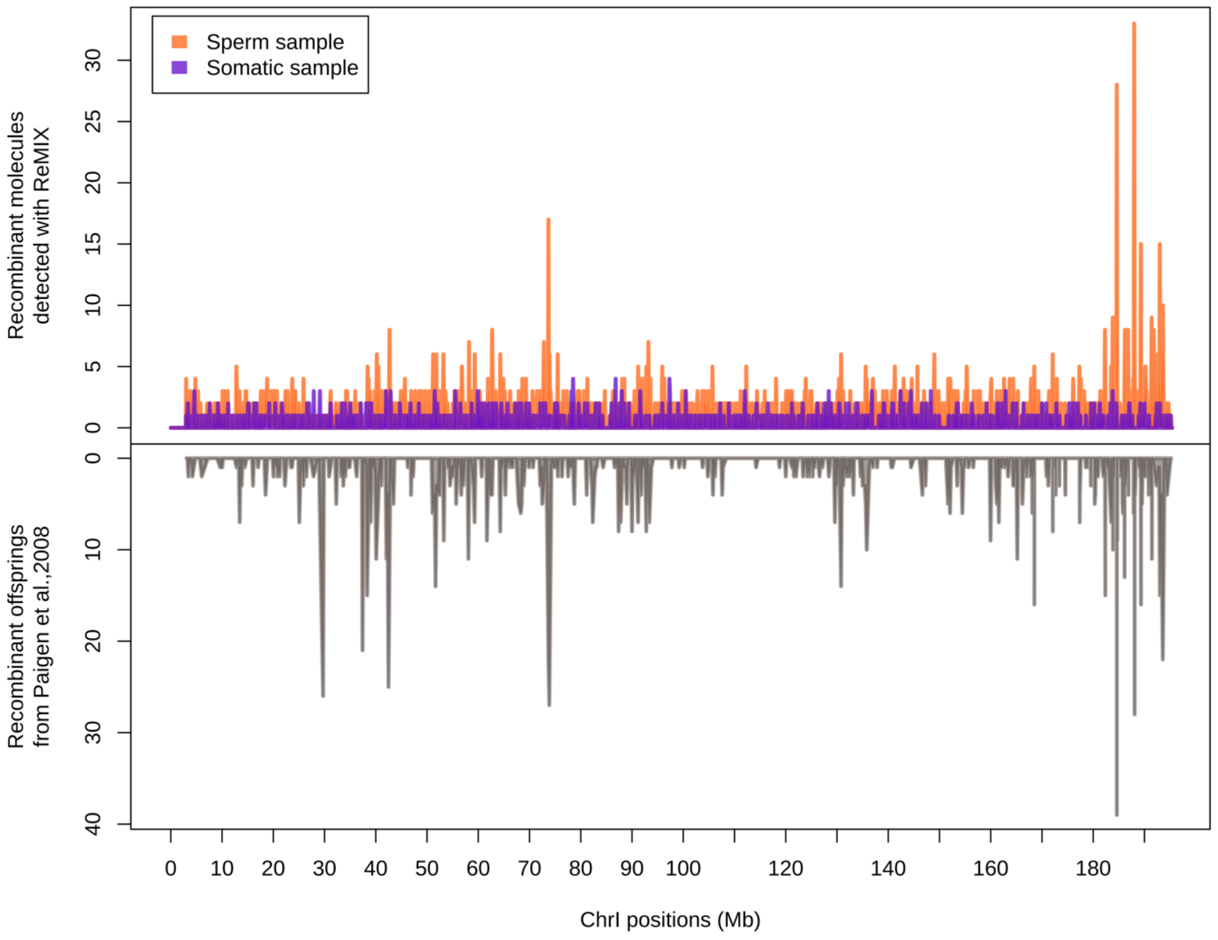

Supplementary Figure 2: ReMIX correctly detects fine-scale recombination variation and hotspots on mouse chromosome 1. The recombination rate determined by ReMIX corresponds well to the rate described in Paigen et al.[1]. The dissimilarity observed in the northern end of the chromosome may be caused by potential sub-strain differences in recombination[2] (C57BL/6Ncrl x CAST/EiJ used in our study and C57BL/6J x CAST/EiJ in Paigen et al.[1].)

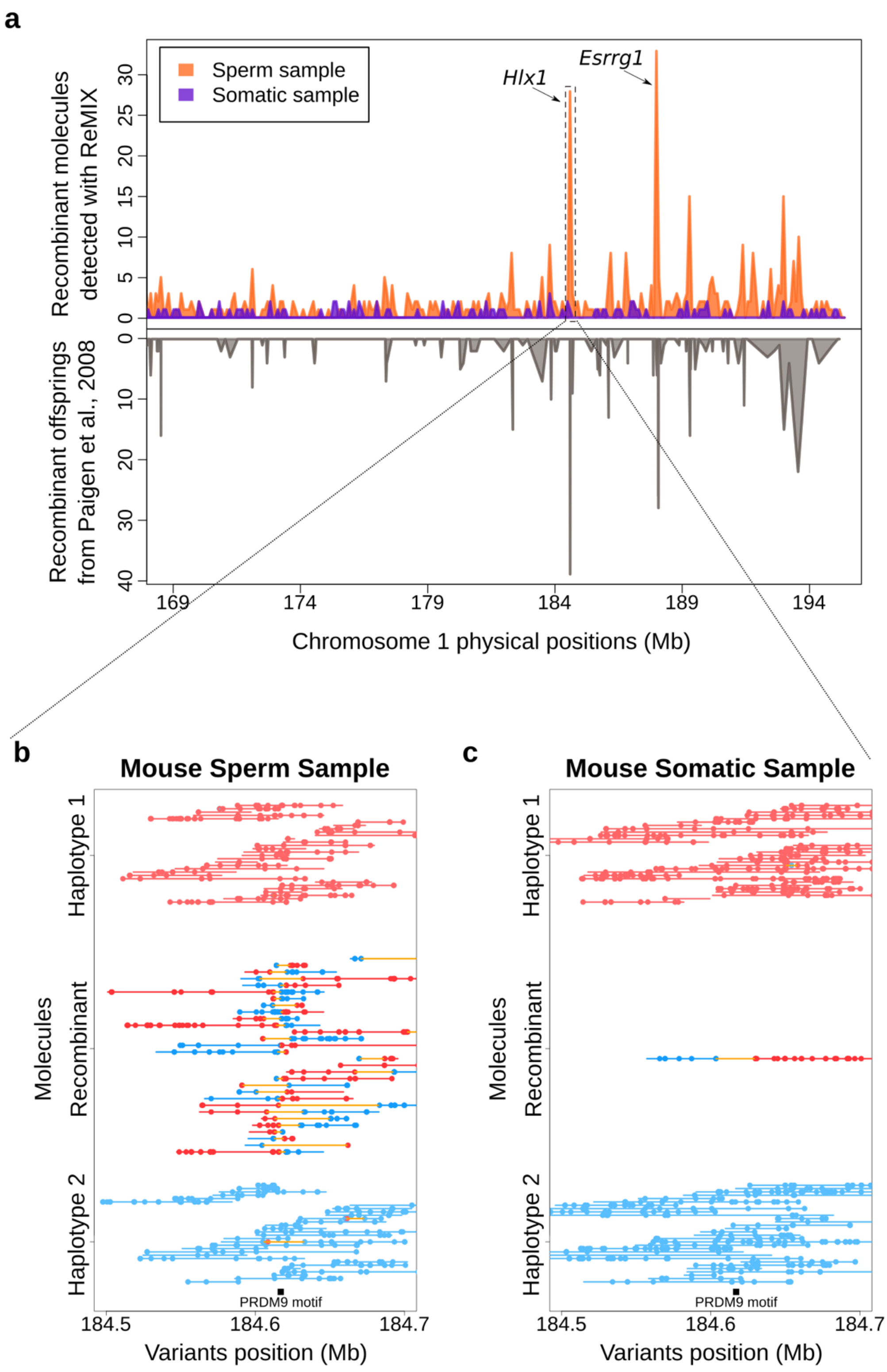

Supplementary Figure 3: ReMIX correctly detects fine-scale recombination variation and hotspots on mouse chromosome 1. **(a)** The recombination rate on the south end of chromosome 1 (169-195.4Mb, mm10), determined by ReMIX corresponds well to the rate described in Paigen et al.[1]. **(b)** The three types of molecules identified by ReMIX in the sperm sample in the region of a well-known recombination hotspot (Hlx1[1,3]). PRDM9 plays a role in initiating crossovers at the Hlx1 hotspot and has a DNA binding motif (black bar) located near the midpoint of the detected recombinant molecules. **(c)** The corresponding region for somatic tissue in which ReMIX identified a recombinant molecule due to mitotic recombination or barcode collision.

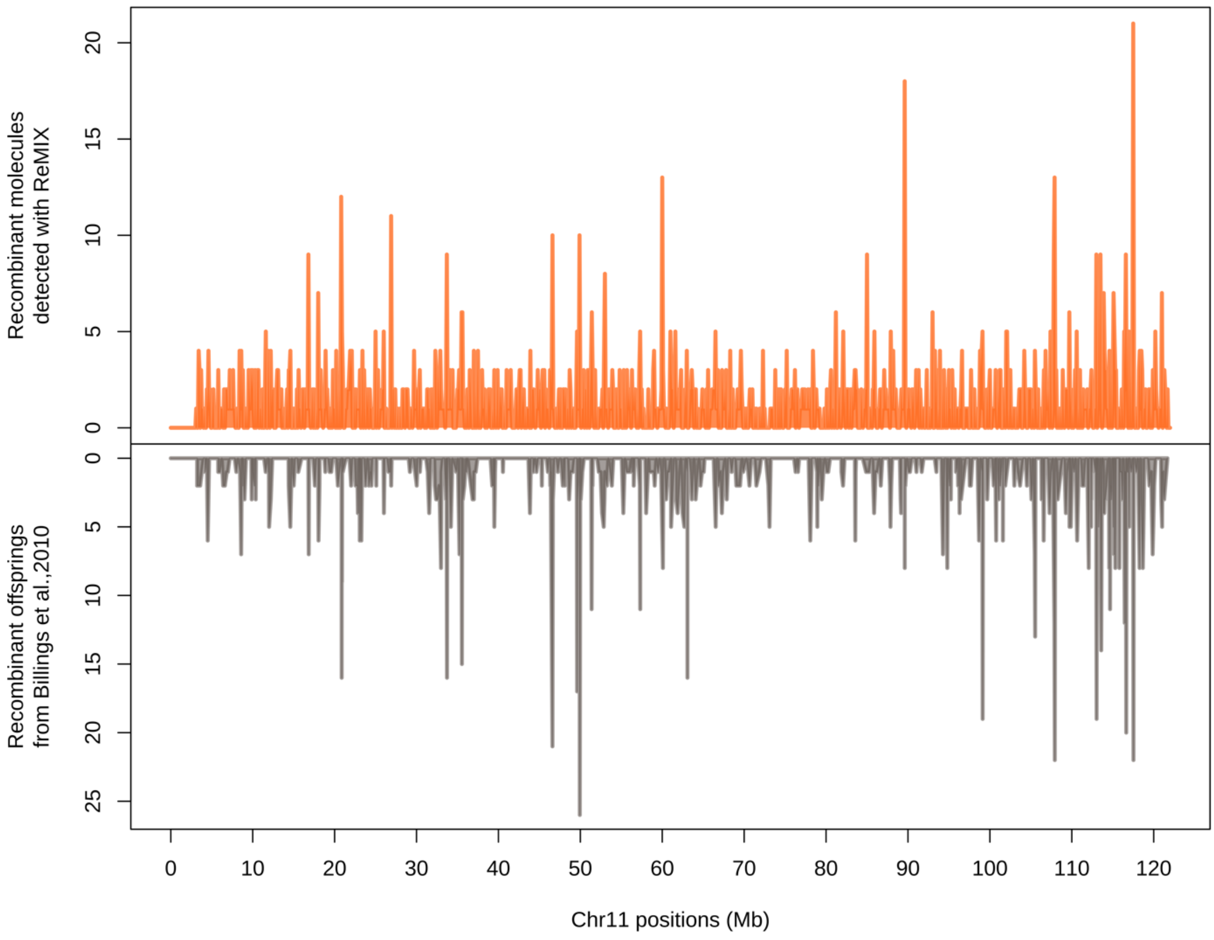

Supplementary Figure 4: ReMIX correctly detects fine-scale recombination variation and hotspots on mouse chromosome 11. The recombination rate determined by ReMIX corresponds well to the rate described in Billings et al. [4].

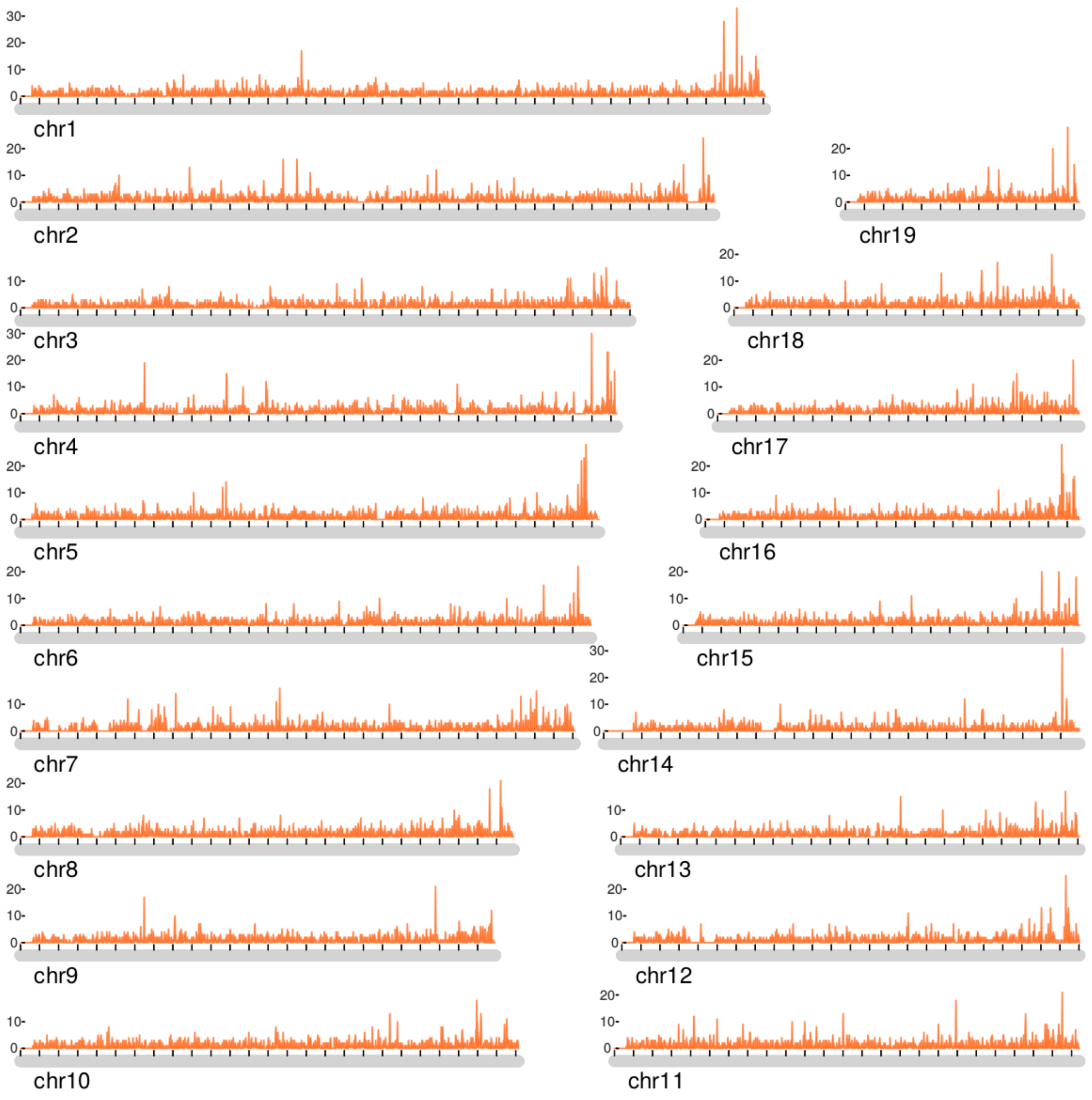

Supplementary Figure 5: ReMIX results on the mouse autosomes. Consistent with previous studies[5], ReMIX reveals recombination crossovers are enriched towards the distal ends of chromosomes in male germline.

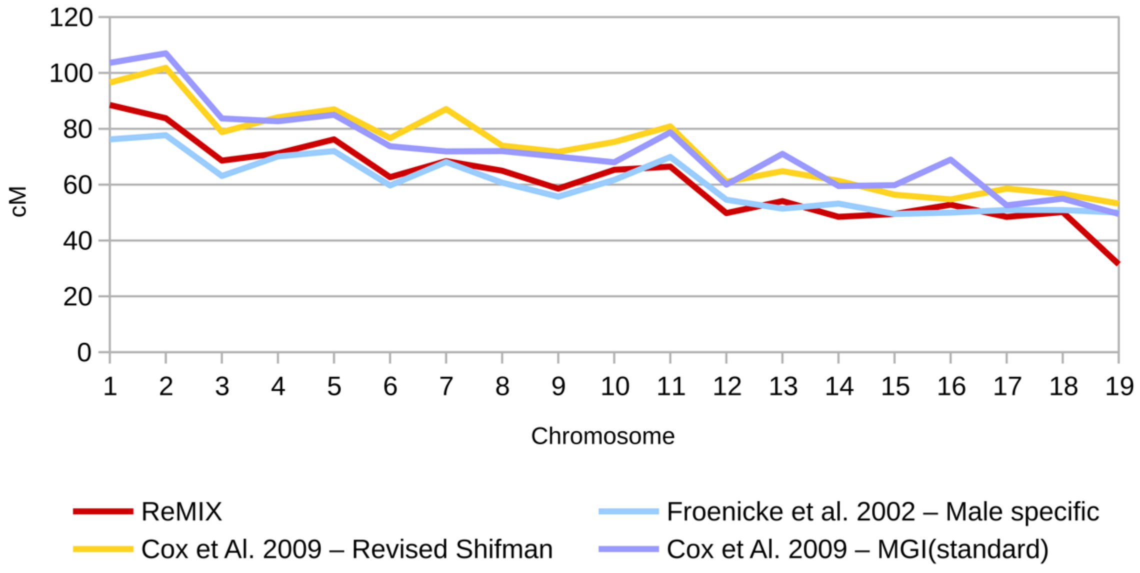

Supplementary Figure 6: Genetic map length comparison between previous studies[6,7] analyzing various mouse strains and ReMIX results on the mouse genome.

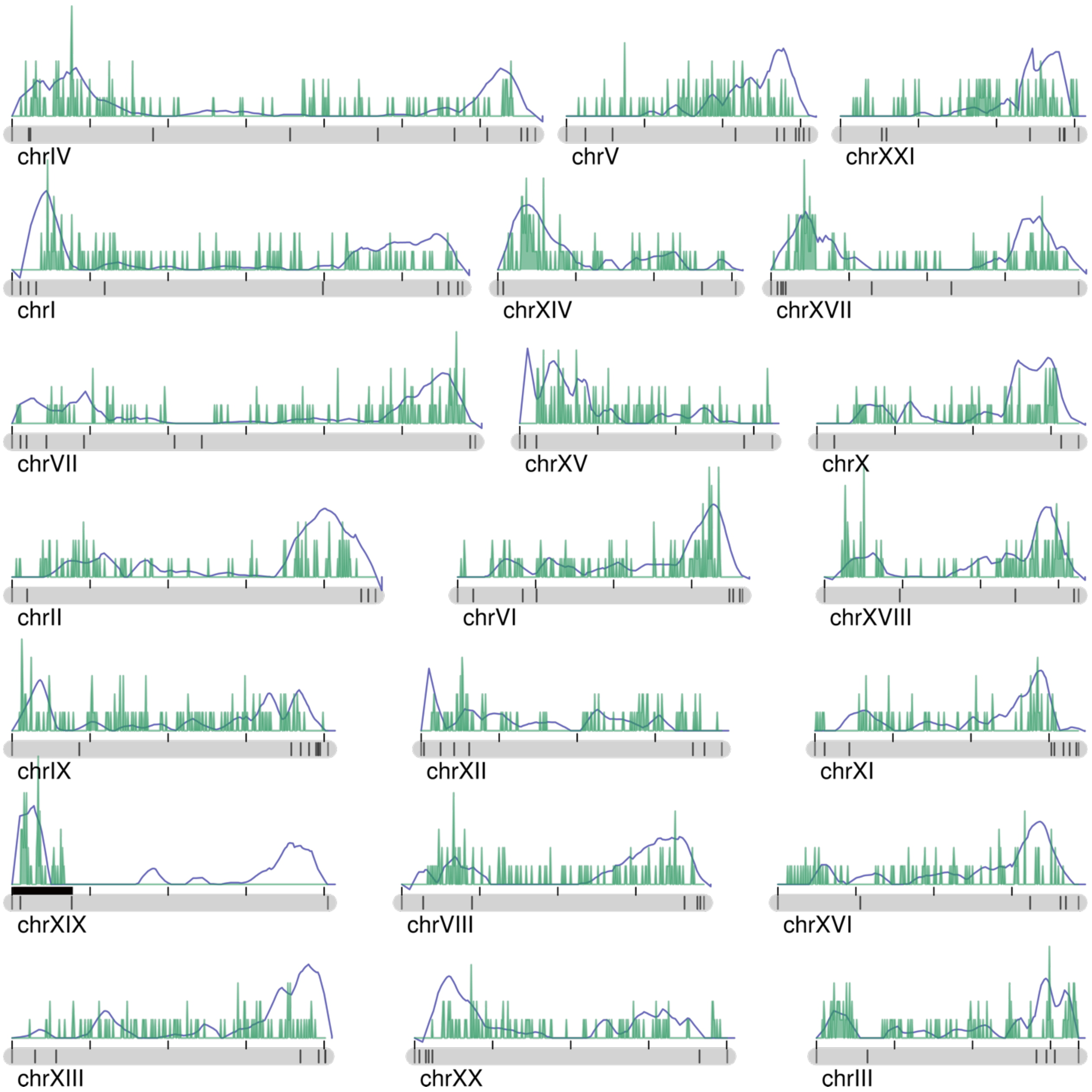

Supplementary Figure 7: Genome graph of recombination events in a male freshwater stickleback with underlying genetic map. The number of crossovers identified by our pipeline is plotted in 50 kb intervals (in green). The genetic map was previously constructed from F2 lab cross population of 282 male and female individuals and 1872 total markers[8] (in blue). Black box on chromosome XIX represents the recombining pseudoautosomal region of the X chromosome. In an XY stickleback male, no recombination is expected in the sex determining region of the X chromosome.

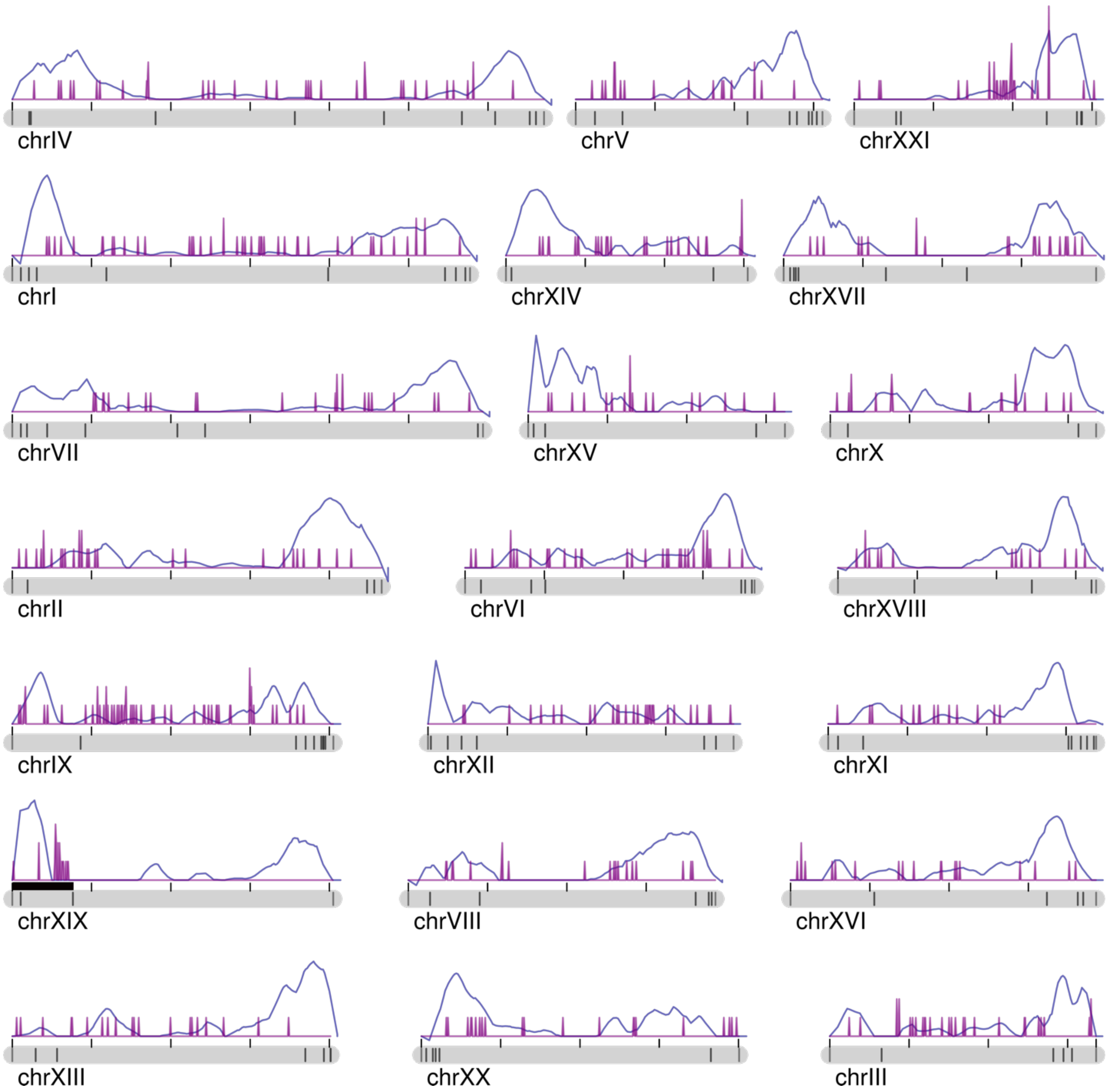

Supplementary Figure 8: Genome graph of recombination events in somatic tissue sample with underlying genetic map for negative control. The number of crossovers identified by our pipeline is plotted in 50 kb intervals (in purple). The genetic map was previously constructed from F2 lab cross population of 282 male and female individuals and 1872 total markers[8] (in blue). For most chromosomes the maximum number of these false positive somatic recombinant molecules in 50 kb windows is 2. As expected, the moderate false positive rate is evenly distributed and does not interfere with the hotspot detection. The false positive rates across chromosomes with elevated levels co-localize with scaffold ends (chromosomes XIV, XIX, and XXI) (black lines on the gray bars) and are likely scaffold assembly errors.

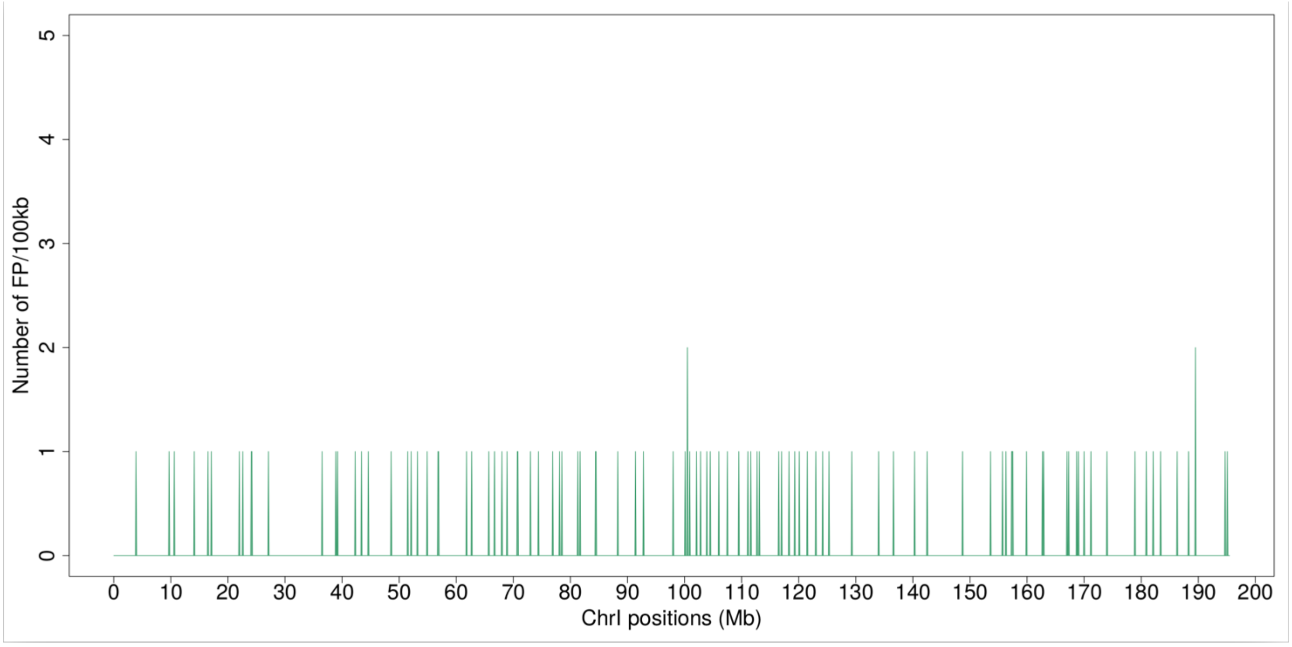

Supplementary Figure 9: Frequency of false positive molecules in 100kb windows in the case of simulating 7 molecules per GEM in the mouse chromosome 1.

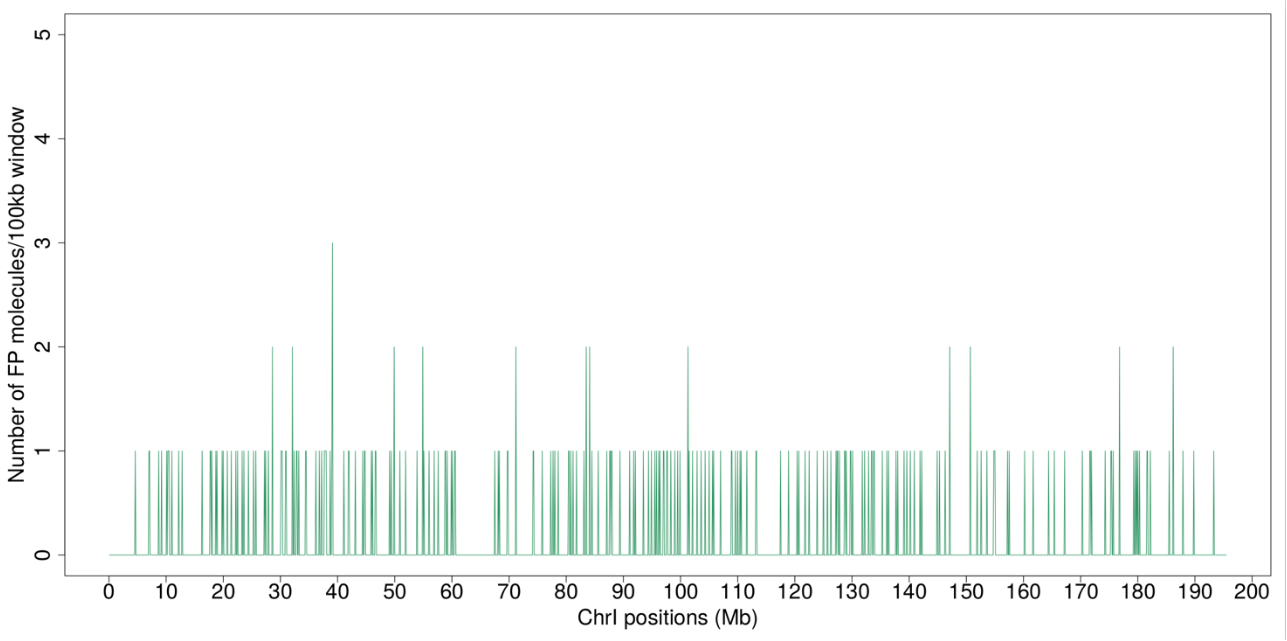

Supplementary Figure 10: Frequency of false positive molecules in 100kb windows in the case of simulating 10 molecules per GEM in the mouse chromosome 1.

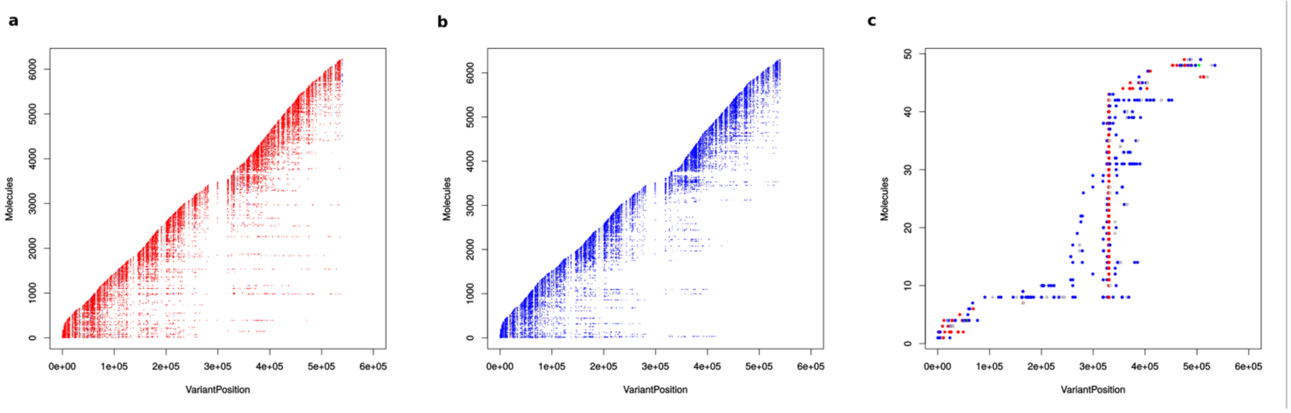

Supplementary Figure 11: The errors in the read alignment and variant calling due to structural variants can generate recombinant-like molecules. (a) Haplotype 1 molecules. (b) Haplotype 2 molecules. (c) Recombinant-like molecules. These errors can cause incorrect variant phasing or barcode collision cases in the structural variantregions. When misplaced reads and a real molecule share the same barcode and are aligned in the same genomic region, the algorithm used for reconstructing the molecules regroups the misplaced reads and the real molecule in a unique molecule. In the case when the misplaced reads and the real molecule originate from opposite haplotypes or when variants are incorrectly phased, the reconstructed molecules appear as if it would span a crossover event and they pile-up wrongly suggest a hotspot region. ReMIX effectively identifies these problematic regions and removes them in the third step of the pipeline.

References for supplementary material

1 Paigen, K. *et al.* The Recombinational Anatomy of a Mouse Chromosome. *Plos Genetics* **4**, e1000119 (2008).

2 Fontaine, D. A. & Davis, D. B. Attention to background strain is essential for metabolic research: C57BL/6 and the international knockout mouse consortium. *Diabetes* **65**, 25-33, (2016).

3 Billings, T. *et al.* DNA binding specificities of the long zinc-finger recombination protein PRDM9. *Genome Biol* **14**, R35 (2013).

4 Billings, T. *et al.* Patterns of recombination activity on mouse chromosome 11 revealed by high resolution mapping. *PLoS One* **5**, e15340 (2010).

5 Liu, E. Y. *et al.* High-resolution sex-specific linkage maps of the mouse reveal polarized distribution of crossovers in male germline. *Genetics* **197**, 91-106 (2014).

6 Cox, A. *et al.* A new standard genetic map for the laboratory mouse. *Genetics* **182**, 1335-1344 (2009).

7 Froenicke, L., Anderson, L. K., Wienberg, J. & Ashley, T. Male mouse recombination maps for each autosome identified by chromosome painting. *Am J Hum Genet* **71**, 1353-1368 (2002).

8 Roesti, M., Moser, D. & Berner, D. Recombination in the threespine stickleback genome - patterns and consequences. *Mol Ecol* **22**, 3014-3027 (2013).
